# Ultra-purified alginate gel implantation decreases inflammatory cytokine levels, prevents intervertebral disc degeneration, and reduces acute pain after discectomy

**DOI:** 10.1038/s41598-020-79958-9

**Published:** 2021-01-12

**Authors:** Katsuro Ura, Katsuhisa Yamada, Takeru Tsujimoto, Daisuke Ukeba, Norimasa Iwasaki, Hideki Sudo

**Affiliations:** 1 Department of Orthopedic Surgery, Faculty of Medicine and Graduate of Medicine, Hokkaido University, N15W7, Sapporo, Hokkaido 060-8638 Japan; 2 Department of Advanced Medicine for Spine and Spinal Cord Disorders, Faculty of Medicine and Graduate of Medicine, Hokkaido University, N15W7, Sapporo, Hokkaido 060-8638 Japan

**Keywords:** Biomaterials, Cartilage, Pain

## Abstract

Lumbar intervertebral disc (IVD) herniation causes severe low back pain (LBP), which results in substantial financial and emotional strains. Despite the effectiveness of discectomy, there is no existing treatment for post-operative LBP induced by progressive IVD degeneration. Two key factors of LBP are intradiscal inflammation, indicated by tumour necrosis factor alpha (TNF-α) and interleukin-6 (IL-6), and sensory nerve ingrowth into the inner layer of the annulus fibrosus, triggered by nerve growth factor/high-affinity tyrosine kinase A (TrkA) signalling. In an animal models of discectomy, the bioresorbable ultra-purified alginate (UPAL) gel with an extremely low-toxicity has been effective in acellular tissue repair. We aimed to investigate whether UPAL gel can alleviate LBP using a rat nucleus pulposus (NP) punch model and a rabbit NP aspirate model. In both models, we assessed TNF-α and IL-6 production and TrkA expression within the IVD by immunohistochemistry. Further, histological analysis and behavioural nociception assay were conducted in the rat model. UPAL gel implantation suppressed TNF-α and IL-6 production, downregulated TrkA expression, inhibited IVD degeneration, and reduced nociceptive behaviour. Our results suggest the potential of UPAL gel implantation as an innovative treatment for IVD herniation by reducing LBP and preventing IVD degeneration after discectomy.

## Introduction

Lumbar intervertebral disc (IVD) herniation is one of the leading causes of low back pain (LBP)^1^, which causes substantial and emotional strain^2^. Discectomy is effective in removing degenerative IVD materials and relieving nerve compression; however, the defect within IVD produced for discectomy may predispose patients to further IVD degeneration, subsequently worsening both clinical outcomes and long-term LBP^3–5^. The treatment of LBP associated with IVD herniation and degeneration after discectomy remains a challenge^6^. To date, no biomaterials that is suitable as a filling material to replace the resected nucleus pulposus (NP) tissue removed after discectomy has been developed. Further, no materials have been shown to be clinically effective in controlling LBP. Therefore, a new treatment that can reduce IVD degeneration and improve pain is needed.

LBP can be classified as acute, subacute, or chronic according to the onset period^7,8^. Acute LBP has an onset period of less than 4 weeks, whereas subacute LBP has an onset period between 4 and 12 weeks^7,8^. The onset period of chronic LBP is more than 12 weeks^7,8^. Intradiscal inflammation is one of the most contributing factors of acute LBP^9,10^. In the chronic-phase, intradiscal inflammation and sensory nerve ingrowth into the deep inner layer of the annulus fibrosus (AF; also as known as “neoinnervation”) contribute to LBP referred as discogenic pain^11^. In herniated IVDs, the upregulation of the expression of several inflammatory factors including tumour necrosis factor (TNF)-α has been observed^12–15^. In addition, higher levels of proinflammatory mediators, such as TNF-α, interleukin-6 (IL-6), and nerve growth factor (NGF) have been found in the IVDs of symptomatic individuals than in those of asymptomatic individuals^10,16^. TNF-α and IL-6 play key roles in pain development by activating specific signalling pathways and upregulating NGF production^17–19^. NGF exerts its effects through the high-affinity tyrosine kinase A (TrkA) and low affinity (p75) receptors^11^. In particular, the NGF–TrkA signalling has been considered as a marker of neurogenesis because it regulates the innervation of peripheral target fields and the complete differentiation of sensory nerves^20^. Therefore, we hypothesised that the inhibition of TNF-α and IL-6 production can inhibit intradiscal inflammation and neoinnervation, which can subsequently relieve acute LBP and discogenic pain after discectomy.

Recently, a study has reported that the implantation of hyaluronic acid (HA) hydrogel suppressed pain behaviour via the downregulation of inflammatory pathways in a rat IVD injury model^21^. It was suggested that the use of biomaterials to compensate for defects in NP tissue may has the potential to downregulate inflammatory pathways after IVD injury. However, HA gel implantation has no proven biomechanical stability and carries risks such as prolapse into the spinal canal. We have previously revealed the reparative effect of the acellular bioresorbable ultra-purified alginate (UPAL) gel, an innovative biomaterial, on damaged tissues in rabbit and sheep discectomy models^22^. In both models, UPAL implantation resulted in reduced IVD degeneration as evidenced by histological and magnetic resonance imaging (MRI) findings, as well as increased Type 2 collagen expression on immunohistochemistry compared to discectomy alone^22^. Furthermore, its implantation increased the proportion of GD2/Tie2 double-positive cells, a marker of medullary progenitor cells, in a rabbit model^22^. UPAL gel is highly purified with reduced endotoxicity (< 1/10,000 compared to conventional alginate) and has many applications including use in animal models^23–25^. Furthermore, UPAL gel can conform to defects of various shapes without the need to cover or suture the AF using CaCl_2_ surface coverage on AF for rapid curing of the alginate gel, thereby potentially reducing the risk of material extrusion^22^. Currently, we are conducting a first-in-human clinical trial involving implantation of the UPAL gel following discectomy^26^. UPAL gel implantation after discectomy may reduce early post-operative LBP. However, to date, its pain-relieving effects, as well as underlying molecular mechanisms, have not been investigated in animal studies.

Therefore, in this study, we aimed to assess whether UPAL gel implantation inhibits inflammation, neoinnervation, and IVD tissue degeneration in rat and rabbit discectomy models, and whether it reduces the presence of nociceptive behaviour in the rat discectomy model. Because we have previously used a rabbit model^22^, we have also adopted the said model here to study under the same conditions as in previous studies. However, there are few established methods for analysing pain-related behaviours in rabbits; thus, we supplemented this study with a rat model, which has already been used to investigate inflammatory cytokines and pain-related behaviors in IVDs^21^. The present findings indicate that UPAL gel implantation is a novel treatment for IVD herniation that is useful for not only preventing inflammatory mediator production and IVD tissue degeneration after discectomy but also reducing post-operative LBP. We believe that the present study reveals the efficacy of UPAL gel implantation in reducing pain after discectomy and shows that UPAL gel implantation can be an innovative treatment for diseases such as IVD herniation.

## Materials and methods

### Animals

**Figure 1:**
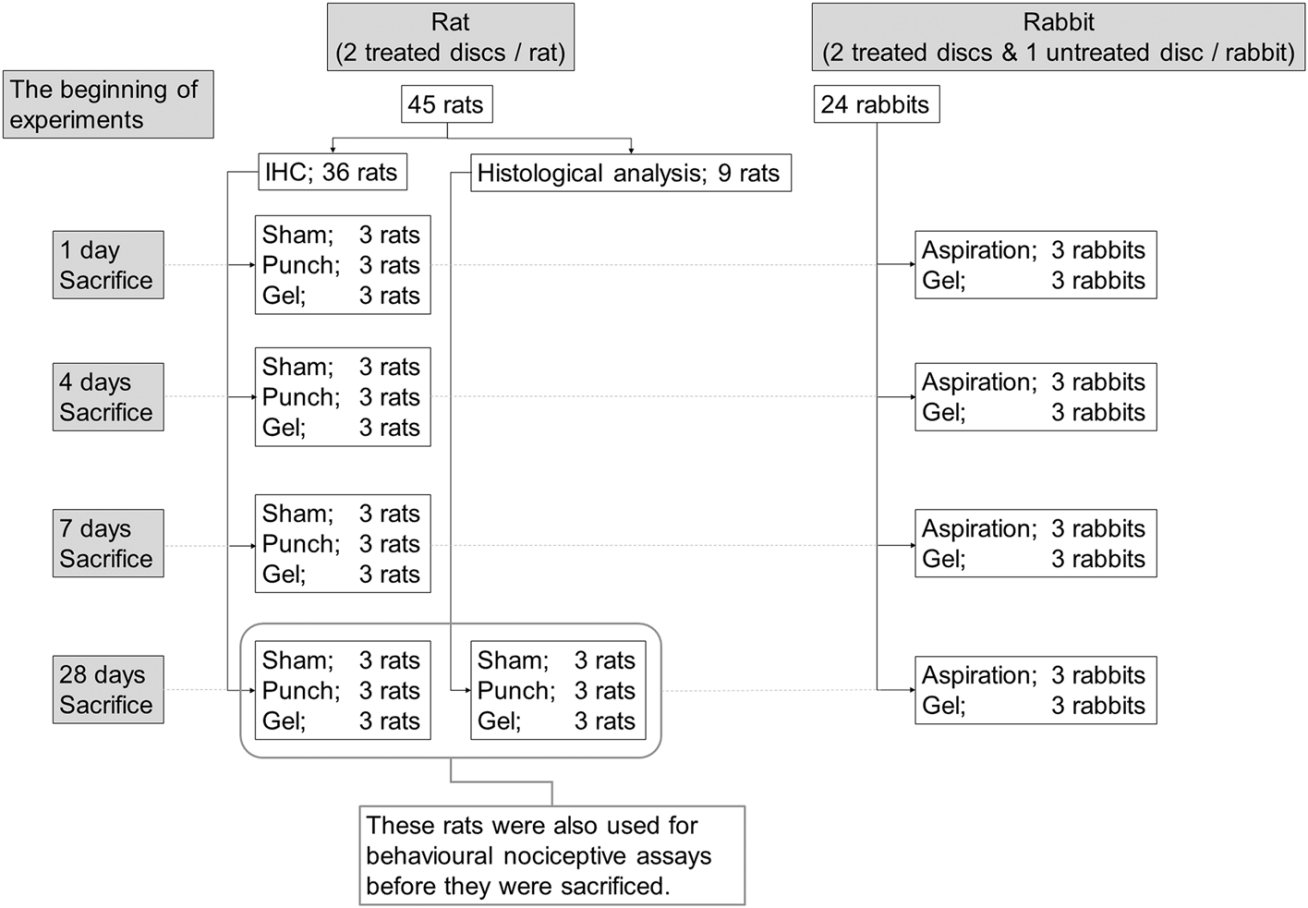
A scheme showing experimental set-up, number of animals, treatment groups, number of treated discs, time points, and analysis.

All animal procedures were performed in accordance with the ARRIVE guideline and National Institutes of Health guide for the care and use of Laboratory animals, and the study was approved by the Institutional Animal Care and Use Committee of Hokkaido University (17-0122). Figure 1 shows the outline of this study (Fig. 1). Outbred female Sprague–Dawley rats (12 weeks old, 260–300 g, n = 45) and outbred male Japanese white rabbits (20–21 weeks old, 3.2–3.5 kg, n = 24) were purchased from Sankyo Labo Service Corporation (Tokyo, Japan) and acclimated to the vivarium for 7 days before experiment. They were kept in cages at 23 ± 2 °C and 50 ± 10% humidity under standard laboratory conditions with a 12-h light/dark cycle and were allowed unrestricted cage activity and ad libitum access to food and water. Rats were housed three per cage, whereas the rabbits were housed individually. The animals were treated aseptically throughout the experiments and all efforts were made to minimise suffering.

### UPAL gel Implantation in the rat caudal NP punch model

A rat caudal NP punch model was used for immunohistochemistry (IHC) analysis, histological analysis, and behavioural nociception assays according to a previous report, with modifications^21^. Rats were randomly allocated to three groups (n = 3 rats; n = 6 IVDs, in each group) as follows using numbered container method: the sham group (skin incision only), punch group (NP punching only), and gel group (UPAL gel implantation after NP punching). Rats were anaesthetized with isoflurane (5% for induction, inhalation) and a mixture of ketamine and medetomidine (ketamine:medetomidine = 75 mg/kg:0.5 mg/kg, intraperitoneal administration). After ensuring that the rats were deeply anaesthetized by tail pinch with forceps, a longitudinal incision at the coccygeal (Co) 4/5 level was made in the dorsal tail skin and connective tissues. The Co4/5 IVD was identified by counting vertebral bodies as the first IVD between the two vertebral bodies when counting from the base of the tail. Then, the NP of Co 4/5 and 5/6 were injured by puncturing through the AF tissue using a 19-gauge (G) needle (the diameter and depth of puncture were 1 mm and 2 mm, respectively). For the gel group, UPAL gel (400–600 mPa/s, Mochida Pharma Co. Ltd., Tokyo, Japan) was prepared as described previously^22^. Briefly, dry and fibrous UPAL was dissolved in a saline solution (0.9%, 2% w/v, Otsuka Pharmaceutical Co., Tokyo, Japan)^22^, and the solution (4 µL per IVD) was immediately injected into the IVD defect using a micro syringe (Hamilton, Reno, NV, USA) with a 26 G needle after NP punching. Then, 102 mM CaCl_2_ (1 mL) was injected on top of the UPAL solution for gelation. Five minutes later, the surgical site was irrigated with normal saline, and the skin and connective tissues were closed using interrupted suture after the confirmation of gelation by inspection and palpation with forceps. For each rat, Co 4/5 and 5/6 were treated identically, i.e. the gel group received gel in both discs, whereas the punch group was left untreated in both discs. Wounds were examined for signs of inflammation or infection until sacrifice. General health assessments were performed on days 1, 4, 7, 14, and 28 after surgery.

### UPAL gel implantation in the rabbit NP aspiration model

A rabbit NP aspiration model^22^, which was used in our previous report that showed the reparative effect of UPAL gel on damaged tissue of IVD, was used for IHC analysis. A total of 24 rabbits were randomly allocated to aspiration-only group (aspiration group) and UPAL gel implantation group (gel group) using numbered container method. The nonsteroidal anti-inflammatory drug carprofen (5 mg/kg) was administrated subcutaneously 15 min before surgery, and general anaesthesia was induced through an intravenous injection of ketamine (10 mg/kg) and xylazine (3 mg/kg) and maintained with oxygen and air (3.0 L/min) mixed with sevoflurane (2–3%) under spontaneous ventilation. Following the subcutaneous injection of a local anesthetic (1% lidocaine), the spine was located via a retroperitoneal approach. AF punctures were performed using an 18 G needle, and NP tissues were aspirated until the contents could not be withdrawn from the lumbar (L) 2/3 and L4/5 IVDs using a 10 ml syringe. L3/4 was left intact to serve as the control^27–30^. In the gel group, 20 μL of UPAL gel was implanted and 102 mM CaCl_2_ (3 mL) was injected on top of the UPAL solution for gelation. Five minutes later, the surgical site was irrigated with normal saline, and the fascia, connective tissues, and skin were closed using interrupted suture after gelation was confirmed by inspection and palpation with forceps. L2/3 and L4/5 were similarly treated in each animal. The same post-surgical care was performed as in the rat model.

### IHC analysis

IHC analysis of rats and rabbits was performed to detect the levels of TNF-α, IL-6, and TrkA 1, 4, 7, and 28 days after surgery. Rats (n = 3 rats; n = 6 IVDs in each group at each time point) were deeply anaesthetized using isoflurane and euthanized using cervical dislocation. Rabbits (n = 3 rabbits; n = 6 IVDs, in each group at each time point) were euthanized using intravenous overdosed injection of pentobarbital sodium after the intravenous administration of 10,000 IU of heparin^27^.

In the rat model, the operated tail (Co 4/5–Co 5/6) was surgically harvested, and the soft tissue was removed under sterile conditions. In the rabbit model, the operated spine (L2/3–L4/5) was surgically harvested, and soft tissue and vertebral bone were removed from the IVDs under sterile conditions. The harvested IVDs (rat, rabbit) were fixed with 4% (w/v) paraformaldehyde for 48 h at room temperature and embedded in paraffin^21,22,31^. Transverse sections (5 μm thick) of the middle portion of the IVD were obtained. The decalcification method was not needed in both rat and rabbit IHC analyses. After deparaffinization in xylene, the sections were incubated with proteinase K (Dako, Agilent Technologies, Santa Clara, CA, USA) for 15 min at 37 °C. Following peroxidase blocking with 1% (w/v) H_2_O_2_ in methanol for 30 min at 37 °C, the specimens were incubated with 2% (w/v) bovine serum albumin for 30 min at room temperature before overnight incubation at 4 °C with the following primary antibodies; for the rat model, an anti-TNF-α mouse monoclonal antibody (1:500, Abcam, Cat# ab220210), anti-IL-6 mouse monoclonal antibody (1:500, Abcam, Cat# ab9324, RRID:AB_307175), and anti-TrkA rabbit monoclonal antibody (1:200, Abcam, Cat# ab86474, RRID:AB_1951357); for the rabbit model, an anti-TNF-α mouse monoclonal antibody (1:1000, NBP2-34372, Novus Biologicals, Centennial, Colorado, US), anti-IL-6 mouse monoclonal antibody (1:200, Cloud-Clone Corp., Cat# MAA079Rb21, Houston, Texas, US), and anti-human NTRK1/TrkA rabbit polyclonal (IgG) antibody (1:1000, LSBio, Cat# aa342-361, Seattle, US). Then, the specimens were stained using HISTOFINE Fast Red II (1:50, substrate kit for alkaline phosphatase, Nichirei Bioscience Inc., Tokyo, Japan) for TNF-α analysis, HistoGreen Substrate kit for Peroxidase (1:25, substrate kit for peroxidase, Cosmo Bio Co., Ltd., Cat# E109, Tokyo, Japan) for IL-6 analysis, and HISTOFINE DAB (1:25, substrate kit for peroxidase, Nichirei Bioscience Inc., Tokyo, Japan) for TrkA analysis. Specimens that were not treated with a primary antibody were used as negative controls (Supplementary Fig. 1). The nuclei were counterstained: hematoxylin was used for samples with TNF-α or TrkA staining, and nuclear fast red was used for samples with IL-6 staining. One section was evaluated per disc. Cells positive for TNF-α, IL-6, or TrkA were separately counted in five independent, randomly selected fields using a light microscope (magnification; 40 × , Olympus, Tokyo, Japan)^32^, and the number of positive cells in each staining was calculated as a percentage of the number of total NP or AF cells in each of the NP or AF tissues. All image assessments were performed by two independent blinded observers. Each observer completed three evaluations for one specimen, and the quantitative data are presented as the mean of all individual means in each group.

### Histological analysis

Histological analysis of rats was performed to evaluate IVD degeneration after IVD injury 28 days after surgery. Rats (n = 3 rats; n = 6 IVDs in each group) were euthanized in the same method as the IHC. IVDs harvested in the same method as the IHC were fixed with 4% (w/v) paraformaldehyde for 48 h, decalcified in Kristensen’s decalcifying solution for 2 weeks, washed for 24 h under running tap water, and paraffin-embedded^21^. Mid-sagittal sections (5 μm thick) of rat IVDs were obtained and stained with hematoxylin and eosin (H&E), safranin O (SO), or Alcian blue (AB) to evaluate the histological score^33^. Briefly, H&E staining assessed the structure of cells and tissues; SO, tissue structure and extracellular matrix (ECM); and AB, the ECM. One section was evaluated per disc.

The semiquantitative histological grading system devised by Rutges et al.^33^ for IVD degeneration was used. This classification evaluates the NP and AF anatomical structures visualized by-H&E and SO, resulting in six categories; endplate (H&E), AF morphology (H&E/SO), boundary between AF and NP (H&E/SO), cellularity of NP (H&E), NP matrix (H&E), NP matrix staining (SO)^33^. Each category was scored from zero (nondegenerative characteristics) to two (severe degenerative characteristics). Although unrelated to the scoring, AB staining was also performed for the purpose of assisting in the evaluation of ECM in accordance with Rutges et al.^33^. The total sum of the scores ranging from 0 (healthy IVD) to 12 (entirely degenerated IVD) was determined. All image assessments were performed by two independent blinded observers (magnification; 20 ×). Each observer completed three evaluations for one specimen, and the quantitative data are presented as the mean of all individual means in each group.

### Behavioural nociception assays

A total of 18 rats were used in the behavioural nociception studies; rats sacrificed 28 days after surgery for IHC (n = 3 rats for each group) and histological analysis (n = 3 rats for each group). In all behavioural studies, the rats were habituated to the test environment for 20 min both 24 h before and immediately before the tests. All behavioural tests were performed by the same blinded observer. All rats underwent the Hargreaves, von Frey, and tail-flick tests. The mean of the multiple measurements was calculated for each rat and the means for each group were averaged to generate the presented quantitative data (n = 6 rats for each group).

#### Hargreaves test

The Hargreaves test was performed 2 days before the surgery (day –2) and 2, 7, 14, and 27 days after surgery using a Hargreaves apparatus (Ugo Basile Biological Instruments, Gemonio, Italy)^21^. The rats were placed into individual chambers on top of a glass plate (Ugo Basile Biological Instruments). An infra-red beam, as a thermal stimulus was applied to the ventral surface of the tail on the side opposite the skin incision. The withdrawal latency to the thermal stimulus was then recorded. The intensity of the beam was set to 50% of the maximum output. A cut-off time of 20 s was imposed to prevent tissue damage. On each day, the rats underwent four trials with a break of at least one minute between trials.

#### von Frey test

The von Frey test was performed 2 days before surgery (day –2) and 2, 7, 14, and 27 days after surgery using a dynamic plantar aesthesiometer (Ugo Basile Biological Instruments). The rats were placed into individual compartments with wire mesh floors and lids with air holes (Ugo Basile Biological Instruments). The filaments (0.5 mm in diameter, nickel-titanium alloy) were applied to the ventral surface of the base of the tail. A linear increase in force from 0 to 5 g was applied over 10 s, after which the force remained constant at 5 g for 30 s^34^. The tail withdrawal latency was recorded as the sensory threshold when the rats showed any of the following nociceptive reactions; flicking, licking, withdrawing, or shaking of the base of the tail. The test was repeated five times for each rat at 10-s intervals.

#### Tail-flick test

In order to prevent tissue damage from excessive thermal stimulation, the tail-flick test was performed 1 day before surgery (day –1) and 3, 8, 15, 28 days after surgery using a heat flux radiometer (Ugo Basile Biological Instruments). Each rat was habituated for 10 min, with only the tail placed onto the apparatus, whereas remaining body was covered with a towel. A beam, as a thermal stimulus, was applied on the ventral surface of the tail 5 cm from the distal end of the tail^23^. The tail-flick latency to the thermal stimulus was then recorded. A cut-off time of 20 s was imposed to prevent tissue damage. On each day, the rats underwent four trials with a break of at least 15 s break between trials.

### Statistical analysis

Sample sizes for the quantitative data were determined based on previous reports^21,22^. The type of distribution for all tested variables was determined using the Shapiro–Wilk test. The critical level of significance was assumed *P* = 0.05. For measurable variables, arithmetic means, standard deviations (SD), medians as well as the range of variability were calculated. In the IHC, histological analyses, and behavioural assays, Kruskal–Wallis test was used for multiple group comparisons. All Kruskal–Wallis test results were further evaluated with the Steel–Dwass test. Randomization of samples was carried out, and the experimenters were blinded for all experiments. All statistical computations were performed using the JMP Pro v13.0 statistical software (SAS Institute, Cary, NC, USA). Statistically significance was considered at *P* < 0.05.

## Results

### UPAL gel implantation inhibits inflammatory cytokine production after IVD injury

**Figure 2:**
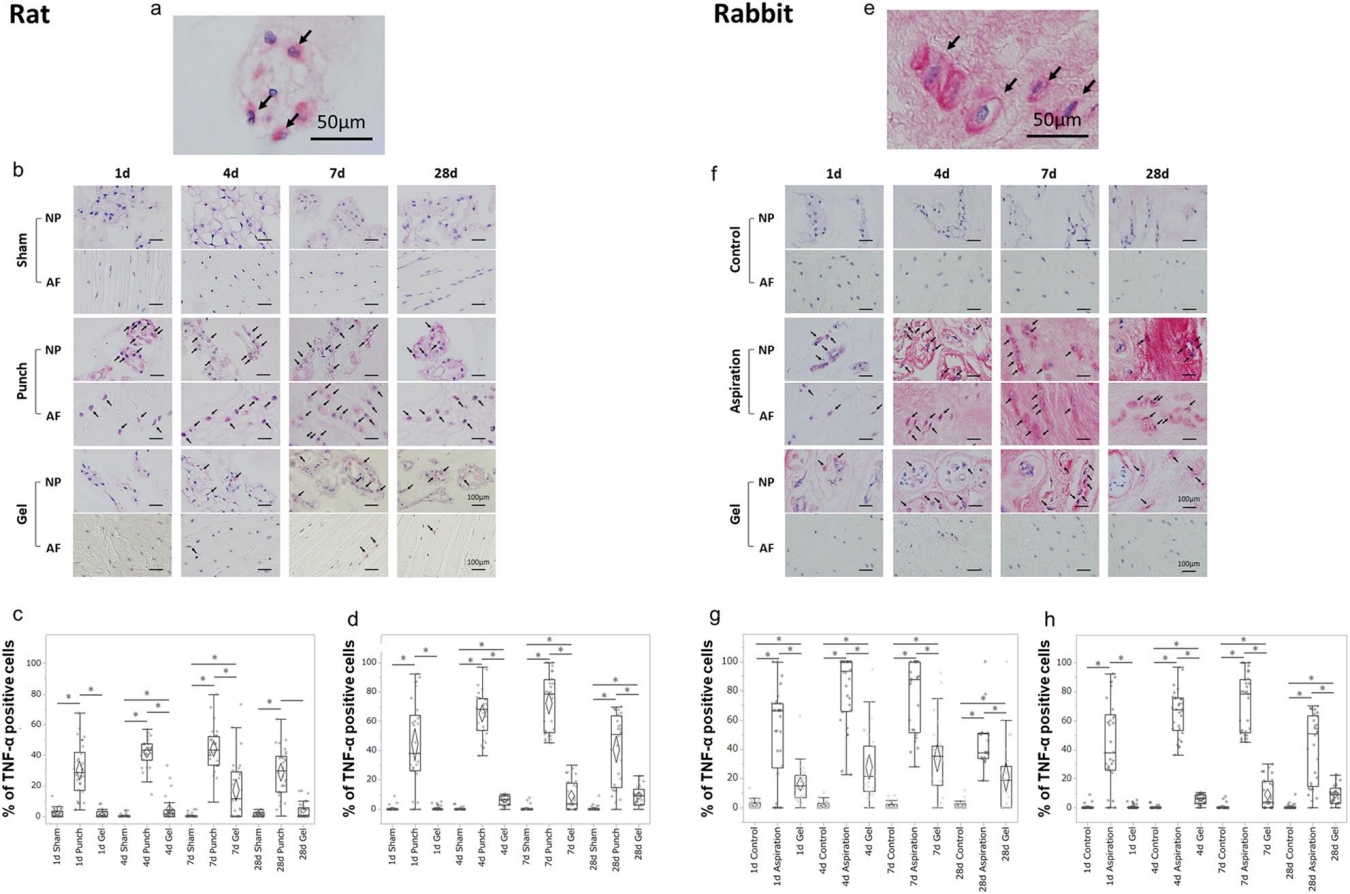
UPAL gel inhibits tumour necrosis factor alpha (TNF-α) production in rat and rabbit intervertebral disc (IVD) degeneration models. The rat nucleus pulposus (NP) punch (**a**–**d**) and rabbit NP aspiration (**e**–**h**) models were used for immunohistochemical (IHC) analysis to detect the TNF-α levels 1, 4, 7, and 28 days after surgery. The number of TNF-α-positive cells in each staining was calculated as a percentage of the number of total NP or annulus fibrosus (AF) cells in five independent, randomly selected fields per disc. TNF-α-positive cells are stained red, and nuclei was stained purple. (**a**) Representative image of TNF-α-positive NP cells in the rat model (allows). Scale bar, 50 µm. (**b**) Representative IHC staining for TNF-α 1, 4, 7, and 28 days after surgery in rat NP and AF tissues. Arrows indicate TNF-α-positive cells. Scale bar, 100 µm. Percentage of TNF-α-positive cells in rat NP tissue (**c**) and AF tissue (**d**). (**e**) Representative image of TNF-α-positive NP cells in the rabbit model (arrows). Scale bar, 50 µm. (**f**) Representative IHC staining for TNF-α 1, 4, 7, and 28 days after surgery in the rabbit NP and AF tissues. Arrows indicate TNF-α-positive cells. Scale bar, 100 µm. Percentage of TNF-α positive cells in rabbit NP tissue (**g**) and AF tissue (**h**). The percentages of TNF-α-positive NP and AF cells in the gel group were significantly lower than those in the punch or aspiration group at each time point in the rat or rabbit model, respectively (*P < 0.05). The boxes represent the median and the interquartile range, with the vertical lines showing the range. The rhombus shape represents the 95% confidence interval (at each time point, n = 3 rats; n = 6 IVDs in each group).

**Figure 3:**
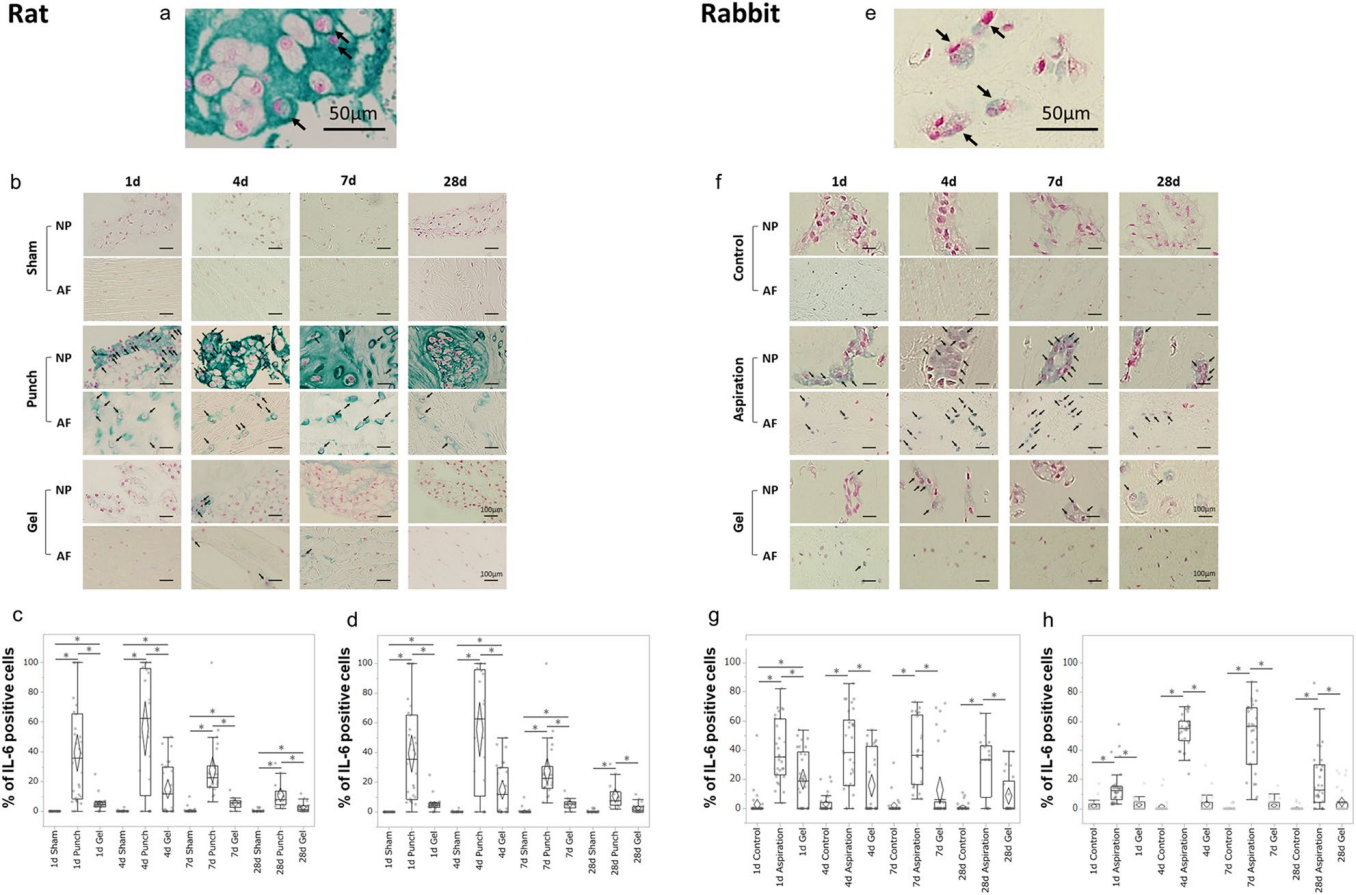
UPAL gel inhibits interleukin-6 (IL-6) production in rat and rabbit intervertebral disc (IVD) degeneration models. The rat nucleus pulposus (NP) punch (**a**–**d**) and rabbit NP aspiration (**e**–**h**) models were used for immunohistochemical (IHC) analysis to detect the IL-6 levels 1, 4, 7, and 28 days after surgery. The number of IL-6-positive cells in each staining was calculated as a percentage of the number of total NP or annulus fibrosus (AF) cells in five independent, randomly selected fields per disc. IL-6-positive cells are stained green, and nuclei was stained red. (**a**) Representative image of IL-6-positive NP cells in the rat model (allows). Scale bar, 50 µm. (**b**) Representative IHC staining for IL-6 1, 4, 7, and 28 days after surgery in rat NP and AF tissues. Arrows indicate IL-6-positive cells. Scale bar, 100 µm. Percentage of IL-6-positive cells in rat NP tissue (**c**) and AF tissue (**d**). (**e**) Representative image of IL-6-positive NP cells in the rabbit model (arrows). Scale bar, 50 µm. (**f**) Representative IHC staining for IL-6 1, 4, 7, and 28 days after surgery in the rabbit NP and AF tissues. Arrows indicate IL-6-positive cells. Scale bar, 100 µm. Percentage of IL-6 positive cells in rabbit NP tissue (**g**) and AF tissue (**h**). The percentages of IL-6-positive NP and AF cells in the gel group were significantly lower than those in the punch group or aspiration at each time point in the rat or rabbit model, respectively (*P < 0.05). The boxes represent the median and the interquartile range, with the vertical lines showing the range. The rhombus shape represents the 95% confidence interval (at each time point, n = 3 rats; n = 6 IVDs in each group).

To determine whether UPAL gel implantation inhibits inflammation, we first investigated the effects of UPAL gel implantation on inflammatory cytokine production (Figs. 2a–h, 3a–h).

In rats, the percentages of TNF-α-positive NP and AF cells in the punch group gradually increased from day 1 to 7 postoperatively but decreased on day 28 (Fig. 2b–d). Although a similar trend was found in the gel group (Fig. 2b), the percentages of TNF-α-positive NP and AF cells in the gel group were significantly lower than those of the punch group at each time point (punch vs. gel, *P* < 0.01 at each time point, Fig. 2c,d). The percentages of TNF-α-positive NP cells in the gel group were significantly higher than those of sham group at postoperative days 4 and 7 (sham vs. gel, *P* = 0.02 on day 4, *P* < 0.01 on day 7, Fig. 2c). The percentages of TNF-α-positive AF cells in the gel group were significantly higher than those of sham group at postoperative days 4, 7, and 28 (sham vs. gel, *P* < 0.01 on day 4 and 7, *P* = 0.01 on day 28, Fig. 2d). In the rabbit model, the percentages of TNF-α-positive NP and AF cells in the gel group were significantly lower than those in the aspiration group at each time point (aspiration vs. gel, *P* < 0.01 at each time point, Fig. 2f–h). In the gel group, the percentages of TNF-α-positive NP and AF cells were significantly higher than those in the control group at each time point (control vs. gel, *P* < 0.01 at each time point, Fig. 2g,h), except for the percentages of AF cell on day 1.

Regarding the percentages of IL-6-positive NP and AF cells in the punch and gel group in the rat model, the percentages of IL-6-positive NP cells decreased from days 4 to 28 and the percentages of IL-6-positive AF cells decreased from days 7 to 28 (Fig. 3b–d). However, the percentages of IL-6-positive NP and AF cells in the gel group were significantly lower than those in the punch group at each time point (punch vs. gel, NP cells; *P* < 0.01 on day 1, 7, 28, *P* = 0.046 on day 4, AF cells; *P* < 0.01 on day 1, 4, 28, *P* = 0.01 on day 7, Fig. 3c,d). The percentages of IL-6-positive NP and AF cells in the gel group were significantly higher than those of sham group (sham vs. gel, *P* < 0.01 at each time point except for the percentages of NP cells on day 28, NP cells; *P* = 0.02 on day 28, Fig. 3c,d). In rabbits, the postoperative changes in the percentages of IL-6-positive NP and AF cells were similar to those in rats (Fig. 3f), and the percentages of IL-6-positive NP and AF cells in the gel group were significantly lower than those in the aspiration group at each time point (aspirate vs. gel, NP cells; *P* = 0.04 on day 1, *P* < 0.01 on day 4 and 7, *P* = 0.01 on day 28, AF cells; *P* < 0.01 at each time point, Fig. 3g,h). In the gel group, the percentages of IL-6-positive NP and AF cells were not significantly different between the control and gel groups, except for the percentages of NP cells on day 1 (control vs. gel, *P* < 0.01, Fig. 3g,h). These results suggest that UPAL gel implantation decreases the inflammatory response produced by IVD injury in both rat and rabbit models.

### UPAL gel implantation inhibits the upregulation of TrkA expression after IVD injury

**Figure 4:**
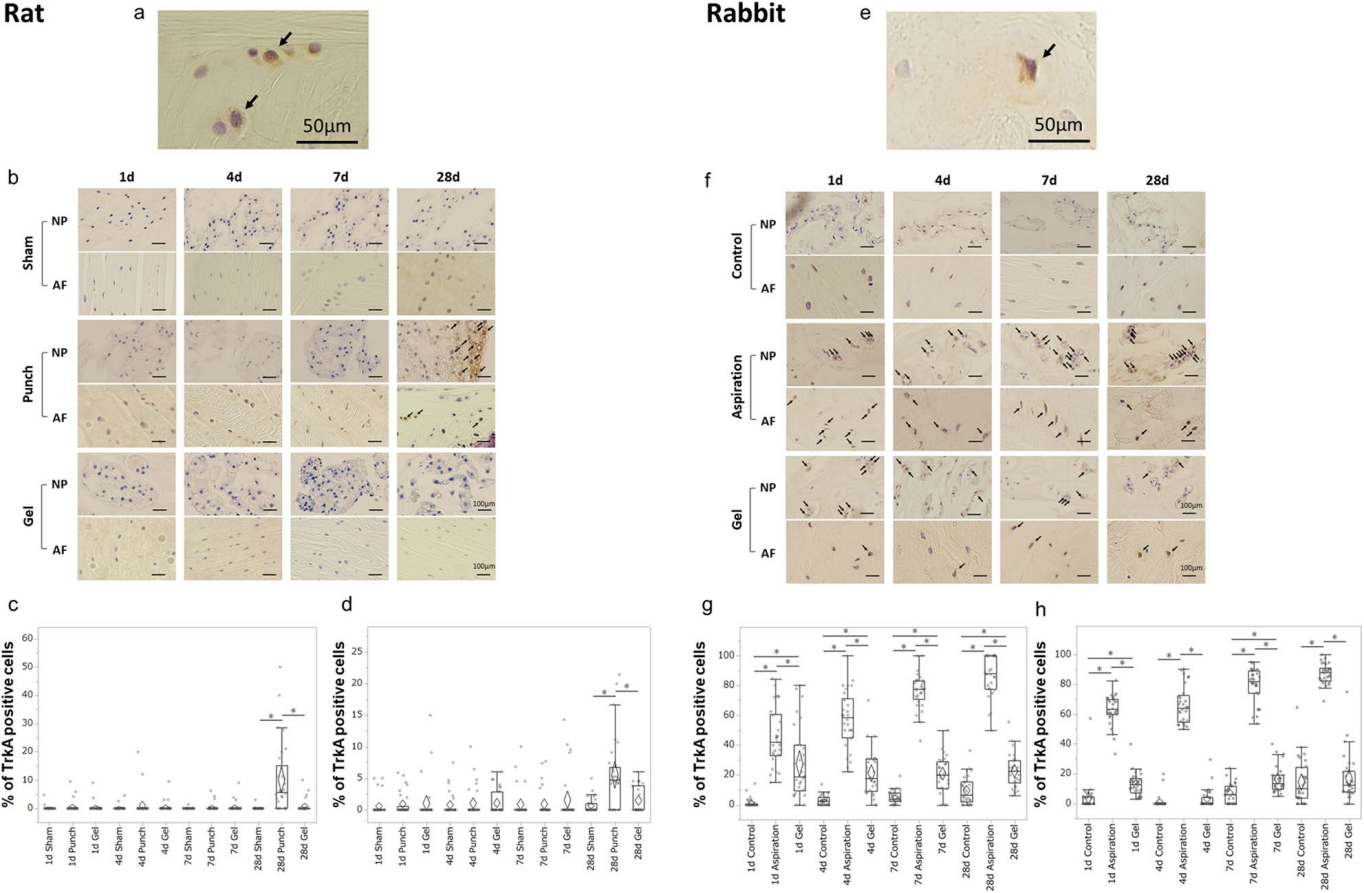
UPAL gel inhibits tyrosine kinase A (TrkA) receptor upregulation in rat and rabbit intervertebral disc (IVD) degeneration models. The rat nucleus pulposus (NP) punch (**a**–**d**) and rabbit NP aspiration (**e**–**h**) models were used for immunohistochemical (IHC) analysis to detect the TrkA levels 1, 4, 7, and 28 days after surgery. The number of TrkA-positive cells in each staining was calculated as a percentage of the number of total NP or annulus fibrosus (AF) cells. TrkA-positive cells are stained brown, and nuclei was stained purple. (**a**) Representative image of TrkA-positive NP cells in the rat model (allows). Scale bar, 50 µm. (**b**) Representative IHC staining for TrkA 1, 4, 7, and 28 days after surgery in rat NP and AF tissues. Arrows indicate IL-6-positive cells. Scale bar, 100 µm. Percentage of TrkA-positive cells in rat NP tissue (**c**) and AF tissue (**d**). (**e**) Representative image of TrkA-positive NP cells in the rabbit model (arrows). Scale bar, 50 µm. (**f**) Representative IHC staining for TrkA 1, 4, 7, and 28 days after surgery in the rabbit NP and AF tissues. Arrows indicate TrkA-positive cells. Scale bar, 100 µm. Percentage of TrkA positive cells in rabbit NP tissue (**g**) and AF tissue (**h**). There was a significant increase in the percentages of TrkA-positive NP and AF cells in the punch or aspiration group 28 days after surgery or at each time point, respectively (*P < 0.05). The boxes represent the median and the interquartile range, with the vertical lines showing the range. The rhombus shape represents the 95% confidence interval (at each time point, n = 3 rats; n = 6 IVDs in each group).

To investigate the effects of UPAL gel on NGF receptor expression in IVD injury, the above rat and rabbit models were used. In each of these models of discectomy, the percentages of TrkA-positive cells were analyzed in IHC 1, 4, 7, and 28 days after surgery and compared among the three groups as same as above (Fig. 4a–h).

In rats, there was no significant difference in the percentages of the TrkA-positive NP and AF cells between the sham and gel groups at each time point (Fig. 4b). The percentages of the TrkA-positive NP and AF cells in all groups showed little change until day 7, and the percentages of the TrkA-positive NP and AF cells in the punch group were increased significantly only on day 28 postoperatively (punch vs. sham, *P* < 0.01, punch vs. gel, *P* < 0.01, Fig. 4c,d). In the rabbit model, the percentages of TrkA-positive NP and AF cells in the aspiration group showed a gradual increase from postoperative day 1 to day 28 (Fig. 4f–h). The percentages of TrkA-positive NP and AF cells in the control and gel groups were nearly unchanged from postoperative day 1 to day 28 (Fig. 4f), and the percentages of TrkA-positive NP and AF cells in the gel group were significantly lower than those in the aspiration group at each time point (aspiration vs. gel, *P* < 0.01, each time point except for the percentages of NP cells on day 1, NP cells; *P* = 0.03 on day 1, Fig. 4g,h). The percentages of TrkA-positive NP and AF cells in the gel group were significantly higher than those in the control group at each time point, except for the percentages of AF cells on day 4 and 28 (control vs. gel, *P* < 0.01, each time point except for the percentages of AF cells on day 4 and 28, Fig. 4g,h). These results suggest that UPAL gel implantation suppresses the intradiscal TrkA receptor expression induced by IVD injury in both rat and rabbit models.

### UPAL gel implantation prevents IVD degeneration in the rat NP punch model

We previously reported the inhibitory effect of UPAL gel implantation on IVD degeneration in the same rabbit IVD aspiration model used in this study^22^. Since the inhibitory effect of UPAL gel on IVD degeneration has not been validated in rats, in this study, we confirmed whether the results we have obtained in rabbits were reproducible in rats.

**Figure 5:**
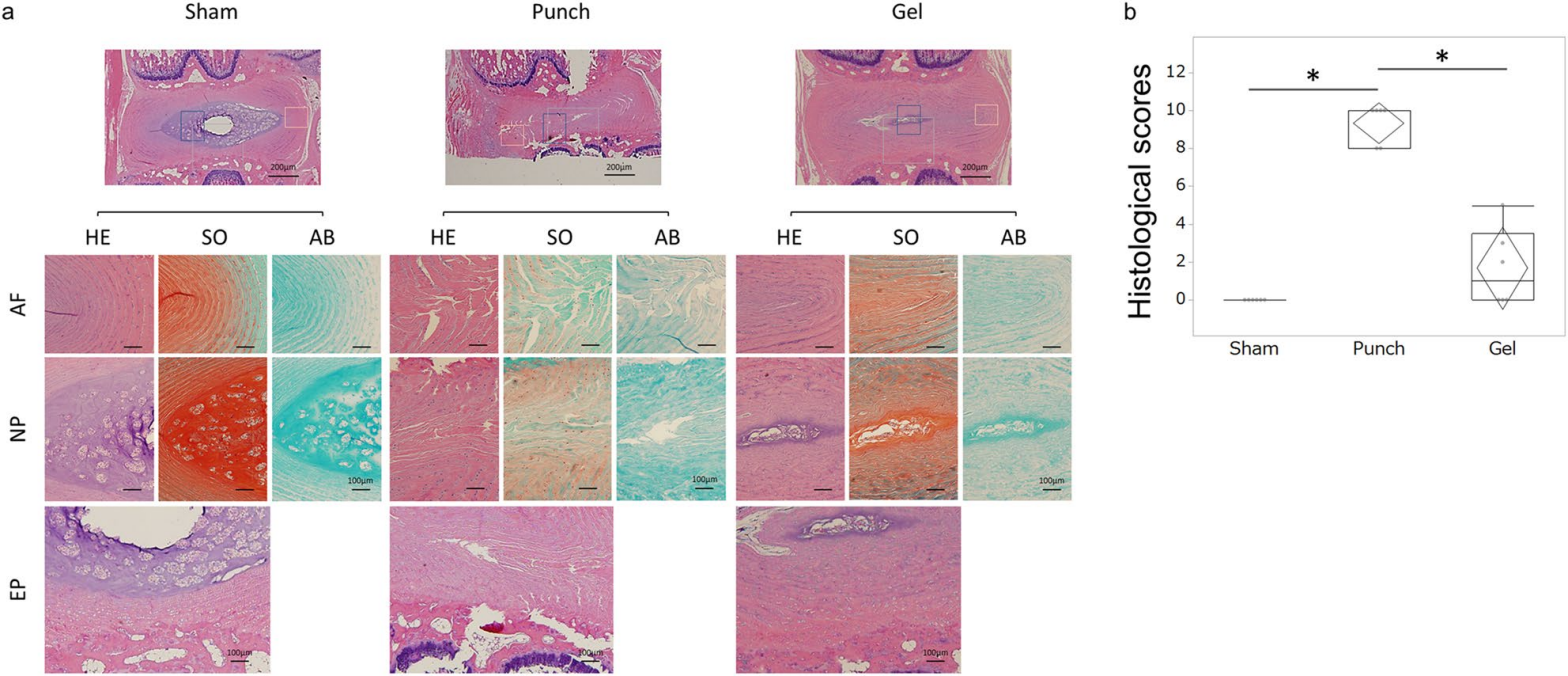
Histological assessment of intervertebral discs (IVDs) showing that UPAL gel prevents IVD degeneration in the rat nucleus pulposus (NP) punch model. Histological analysis was performed to evaluate IVD degeneration after IVD injury 28 days after surgery. Mid-sagittal sections (5 μm thick) of rat IVDs were obtained and stained with Haematoxylin and eosin (H&E), Safranin O (SO), and Alcian blue (AB) to evaluate a histological score (**a**) Representative image of H&E, SO, and AB staining in annulus fibrosus (AF), NP, and endplate (EP) tissues. Scale bar, 100 µm for all except for the top row. 200 µm for the top row images. (**b**) Histological grading (degeneration) score. The score in the sham group was zero. The histological grading (degeneration) score in the gel group was significantly lower than that in the punch group (*P < 0.05). The boxes represent the median and the interquartile range, with the vertical lines showing the range. The rhombus shape represents the 95% confidence interval (at each time point, n = 3 rats; n = 6 IVDs in each group).

Histological evaluation of the IVDs revealed regular endplates, well-organized AF tissue, clear boundaries between the AF and NP, no clusters of NP cells, and well-organized ECM of NP tissue in the sham group (Fig. 5a). In the punch group, severe endplate irregularity, annular rupture, indistinguishable boundary between the AF and NP, mainly clustering of NP cells, complete disorganization and loss of NP matrix, and the chondroid nests of NP tissue were observed 28 days after surgery (Fig. 5a). However, the IVDs in the gel groups showed regular endplates, serpentine lamellae but maintained a half ring structure, mixed NP cellularity, and partly disorganized NP matrix structure (Fig. 5a). The scores for each category are shown in the Supplementary Table 1. AB staining revealed that IVDs in the punch group had faint NP matrix staining, whereas there was intense staining in IVDs in the sham group. In the gel group, IVDs showed reduced NP matrix staining (Fig. 5a, AB columns). Accordingly, the semiquantitative histological IVD degeneration scores^33^ on day 28 in the gel group were significantly lower than those in the punch group (*P* < 0.01, Fig. 5b). There were no significant differences in the histological scores between the gel and sham groups (Fig. 5b). These results suggest that UPAL gel implantation prevents the IVD degeneration after discectomy in the rat models.

### UPAL gel reduces nociceptive behaviour in the rat NP punch model

**Figure 6:**
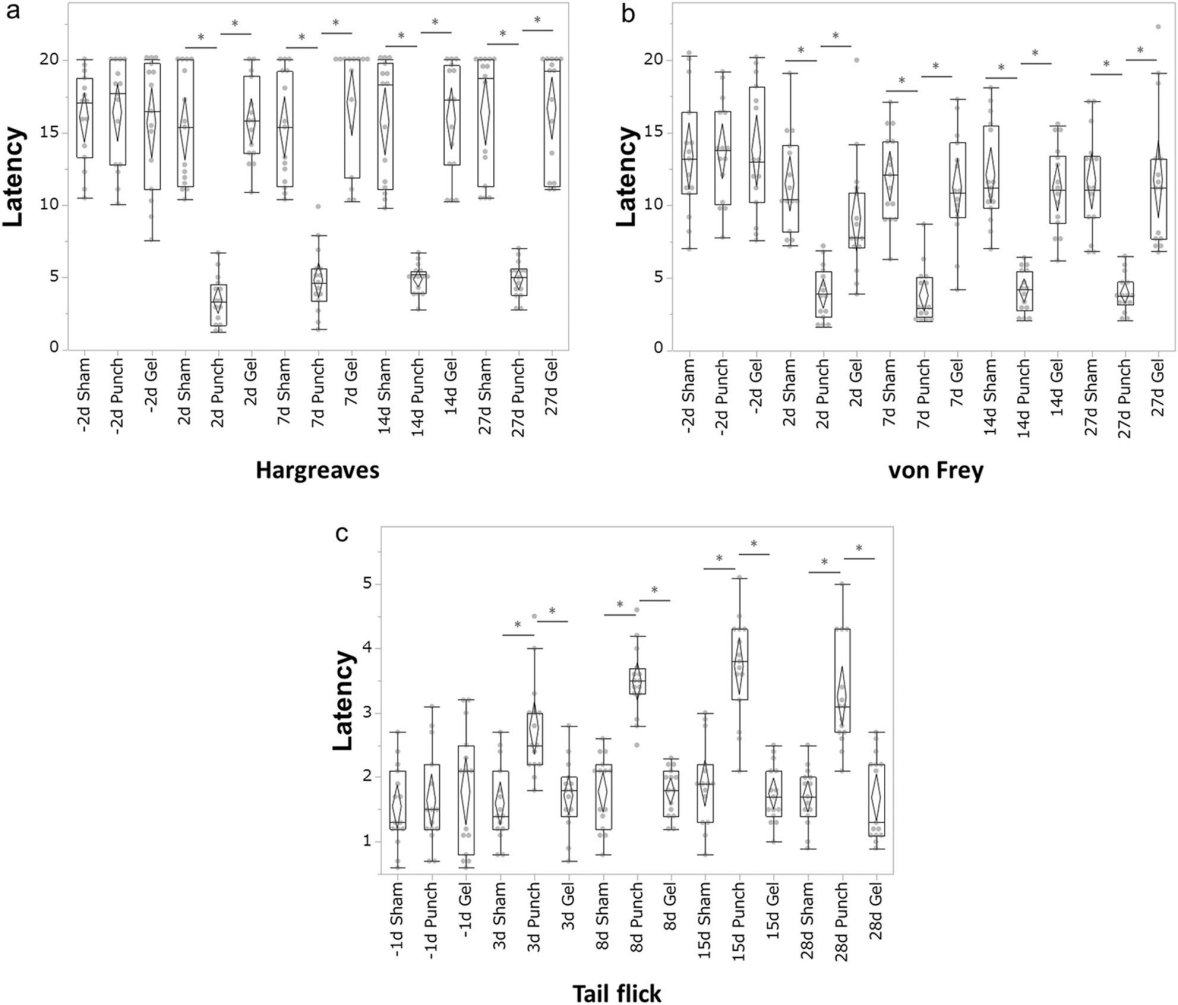
UPAL gel reduces nociceptive behaviour in the rat nucleus pulposus (NP) punch model. The Hargreaves and von Frey tests were performed 2 days before surgery (day −2) and on days 2, 7, 14, and 27 after surgery. The tail-flick test was performed 1 day before surgery (day −1) and on days 3, 8, 15, 28 after surgery. Latency times in the (**a**) Hargreaves test (**b**) von Frey test, and (**c**) tail-flick test. The mean of the multiple measurements was calculated for each rat and then the means for each group were averaged to generated the presented quantitative data. In the Hargreaves and von Frey tests, the withdrawal latency was significantly reduced in the punch group compared to the sham and gel groups from 2 to 27 days postoperatively (*P < 0.05). In the tail flick test, the latency in the punch group increased after surgery and was significantly higher than that in the sham and gel groups at each time point after surgery (*P < 0.05). The boxes represent the median and the interquartile range, with the vertical lines showing the range. The rhombus shape represents the 95% confidence interval (at each time point, n = 6 rats in each group).

Our IHC and histological analysis showed that UPAL gel implantation suppressed the inflammatory response and TrkA expression and inhibited IVD tissue degeneration, which suggested that UPAL gel implantation may reduce pain caused by IVD injury. Therefore, a subsequent assessment of nociceptive behaviour was performed. We analysed the latency time during each quantitative sensory test (Fig. 6a–c).

In the Hargreaves test, the withdrawal latency was significantly reduced in the punch group compared to the sham and gel groups from days 2 to 27 days postoperatively (punch vs. sham, *P* < 0.01 at each time point, Fig. 6a). Similarly, the von Frey test demonstrated that the latency was significantly reduced postoperatively in the punch group compared to the sham and gel groups (punch vs. sham, *P* < 0.01 at each time point, punched vs. gel, *P* < 0.01 at each time point, Fig. 6b). In the tail flick test, the latencies in the sham and gel groups did not change over the course of the experiment, however, the latency in the punch group increased after surgery and was significantly higher than that in the sham and gel groups at each time point after surgery (punch vs. sham or gel, *P* < 0.01 at each time point, Fig. 6c). These results suggest that UPAL gel implantation alleviates the pain induced by IVD injury.

## Discussion

This study aimed to assess whether UPAL gel implantation reduces inflammatory cytokine levels, prevents intervertebral disc degeneration, and reduces acute pain after discectomy in rat and rabbit discectomy models. UPAL gel implantation inhibited TNF-α and IL-6 production and downregulated TrkA receptor expression in both rat and rabbit models. Furthermore, UPAL gel implantation inhibited the IVD degeneration after surgery and reduced the presence of nociceptive behaviours in a rat caudal NP punch model. The present findings indicate the utility of UPAL gel as treatment for IVD herniation that not only prevents IVD tissue degeneration, but also for suppresses inflammation, potentially reduced neoinnervation as suggested by the downregulation of TrkA expression, and alleviates pain induced IVD injury after discectomy.

Previous reports have shown that inflammatory responses are associated with acute-phase LBP^21,35,36^. In the rat caudal NP punch model in the present study, the production of TNF-α and IL-6 in both the NP and AF cells was decreased by UPAL gel implantation. In addition, behavioural analysis of nociceptive stimuli showed that UPAL gel implantation successfully reduced pain-related behaviours. Wuertz et al.^35^ have reported that the high expression of interleukins, including IL-6, and TNF-α plays an essential role in the development of LBP. Lai et al.^36^ have demonstrated that annular puncture followed by TNF-α injection enhanced painful behaviour in vivo. Mohd Isa et al.^21^ revealed that acute-phase response signalling involving IL-6 and IL-1β is one of the pathways contributing to the downstream signalling cascade that activates NF-κB and MAPK, which regulate inflammation and apoptosis. In the present study, UPAL gel implantation suppressed the production of inflammatory cytokines and improved pain-related behaviour, indicating that the inhibition of inflammatory cytokine production by UPAL gel implantation reduces disc pain, as reported for HA hydrogel implantation by Mohd Isa et al.^21^ Using a rabbit model based on Good Laboratory Practice (GLP) standards, we previously showed that UPAL gel exhibited no adverse reaction such as systemic toxicity or injuries, to tissues at implantation sites^22^. Besides, UPAL gel prevented IVD degeneration after discectomy in sheep up to 24 weeks after implantation without any adverse reaction. Since the preclinical proof-of-concept study showed the potential safety and efficacy of UPAL gel^22^, we advanced to the first-in-human clinical trial; we are currently conducting a clinical trial of UPAL gel implantation after discectomy for human lumbar IVD herniation^26^, the findings of which will demonstrate whether the pain-reducing effect of UPAL gel implantation observed in this study is reproducible in humans.

Increased NGF expression induced by pro-inflammatory cytokines, such as IL-1β and TNF-α, and the binding of NGF to TrkA, a high-affinity receptor, promotes neoinnervation into the IVD and local inflammation, thus causing chronic-phase IVD pain (discogenic pain)^17–19^. In this study, the expression of TrkA in the rat caudal NP punch model was postoperatively suppressed in the UPAL gel implantation group up to 28 days. Mohd Isa et al.^21^ have reported that HA gel implantation into injured IVDs of rats reduced NGF–TrkA binding by suppressing in the levels of neurotrophic factors such as NGF, resulting in a reduction in the presence of pain-related behaviours in rats. Lane et al.^37^ showed that anti-NGF antibody treatment significantly decreased knee joint pain in humans compared with placebo, suggesting that inhibition of the NGF–TrkA binding may also reduce discogenic pain. Although nerve ingrowth was not assessed in this study and detection of the NGF receptor is no direct verification of neoinnervation, our results on the downregulation of the TrkA expression within the IVD indicate the reduction in NGF–TrkA binding, which suggests that UPAL gel implantation may potentially suppress or delay subsequent neoinnervation after IVD injury and reduce discogenic pain. In this study, pain behaviour was observed as early as 2 days post operatively till 28 days. UPAL gel implantation could have an alleviating effect on acute-phase IVD pain after discectomy, in addition to a prophylactic effect on discogenic pain.

We also revealed that TrkA expression was suppressed by UPAL gel implantation in both the rat and rabbit models. However, the trend in the rabbit model was different from the results in the rat model; in the rabbit model, TrkA expression was increased in the aspiration group from postoperative day 1 to 28 and its expression was suppressed by UPAL gel implantation. Whereas in the rat model, TrkA expression was increased in the punch group only at postoperative day 28 and was suppressed by UPAL gel implantation. The results for TrkA expression in the rat model were comparable to those reported by Mohd et al.^21^. To the best of our knowledge, there are no reports on TrkA expression in early postoperative rabbit IVDs, and this study may be the first report to investigate this issue. The difference in the timing of the increase in TrkA expression between the rat and rabbit models may be due to species differences.

Degenerative changes in IVD tissues understandably impair disc function, which is important for load bearing, and cause chronic discogenic pain^38^. IVD herniation and discectomy for this disease can cause disc degeneration and subsequent discogenic pain^3–5^. Similar to our previous study using rabbit and sheep IVD degeneration models^22^, we observed that IVD degeneration was inhibited by UPAL gel implantation in the rat NP punch model; for example, SO and AB staining of NP tissues showed better ECM staining in the gel group than that in the punch group, and H&E and SO staining showed less extensive damage in NP and AF tissues in the gel group than the punch group. Therefore, UPAL gel implantation may serve as a prophylactic treatment for discogenic pain by preventing IVD degeneration.

A previous report showed that annular puncture in lumbar IVD evokes mechanical and thermal hyperalgesia in rats^39^. Mohd et al.^21^ have reported a rat caudal NP punch model and analyzed the effect of HA gel implantation on nociceptive behavior using the Hargreaves, von Frey, and tail-flick tests. They reported prolonged latency in the Hargreaves and von Frey tests and a shorter latency in the tail-flick test following HA gel implantation^21^. Mohd et al.^21^ concluded that the injury itself caused a diffuse noxious inhibitory control (DNIC) phenomenon, which induces hypoalgesia in areas other than the painful area because of pain in the injured area, whereas the implantation of HA did not cause DNIC because of the reduced pain in the injured area. In this study, the gel group showed a prolonged latency in the Hargreaves and von Frey tests and a shorter latency in the tail-flick test than those in the punch group, suggesting that UPAL gel implantation reduced the pain in the injured area and suppressed the DNIC phenomenon.

This study has a few limitations. First, no direct causal relationship has been demonstrated between the reduced production of proinflammatory cytokines and reduced pain-related behaviours in the rat NP punch model. The pain-suppressing mechanism of UPAL gel could be considered to be that demonstrated by Mohd Isa et al.^21^, who used HA gel to fill an IVDs defects; the HA gel acts through the reduction of nociception, inhibition of hyperinnervation, and the expression of nociceptive markers via the attenuation of key inflammatory signalling molecules and the modulation of protein regulatory pathways^21^. However, future studies are needed to elucidate molecular mechanisms underlying the observed reduction in inflammation and pain-related behaviours. Second, rabbit models are not suitable for the assessment of pain-related behaviour because there are still no quantitative analysis methods available. However, since the production of proinflammatory cytokines after IVD injury in rabbits showed trends similar to those in rats, it is predicted that pain would also show a similar trend. Last, since behavioural nociception assays for discogenic pain using a filling material after discectomy employ rat tails^21^, we used the tail model. Although most other rat disc pain models are in the lumbar spine^39–41^, there are no significant differences in measuring pain between the lumbar and tail models. However, it is unclear how the present behavioural nociception assays would be relevant to the human lumbar spine which has vastly different anatomy and where pain is not often provoked by direct stimulation.

In conclusion, no suitable biomaterials have been developed as a filling material to replace the resected NP tissue removed after discectomy. Besides, except for those reported in a study by Mohd et al.^21^ and our current study, no materials were experimentally effective in controlling discogenic pain after discectomy. The difference between the two studies is that we previously demonstrated sufficient biomechanical characteristics without material protrusion^22^; both studies show similarity in that both biomaterials prevented inflammation and degeneration at the implantation sites, alleviating pain-induced IVD injury. UPAL gel implantation inhibited the production of TNF-α and IL-6, suppressed TrkA expression, prevented IVD tissue degeneration, and reduced the presence of nociceptive behaviour. Thus, this study showed the potential of UPAL gel implantation an innovative treatment for IVD herniation because it not only prevented for IVD tissue degeneration but also for reduced LBP after discectomy. Following the ongoing first-in-human clinical trial, this novel therapeutic strategy will be planned for clinical application for herniated IVDs.

## Supplementary information

Supplementary Information.

## Data Availability

The datasets generated and/or analysed during the present study are available from the corresponding author on reasonable request.
