# Supplementary material for: Ultra-purified alginate gel implantation decreases inflammatory cytokine levels, prevents intervertebral disc degeneration, and reduces acute pain after discectomy
Source: Sci Rep. 2021 Jan 12;11:638. doi: 10.1038/s41598-020-79958-9 (PMC7804289; doi:10.1038/s41598-020-79958-9)
Supplement: Supplementary file 1 — Supplementary Information. [file 41598_2020_79958_MOESM1_ESM.pdf]

Ultra-purified alginate gel implantation decreases inflammatory cytokine levels, prevents intervertebral disc degeneration, and reduces acute pain after discectomy

Katsuro Ura[1], Katsuhisa Yamada[1*], Takeru Tsujimoto[1], Daisuke Ukeba[1], Norimasa Iwasaki[1], Hideki Sudo[2*]

[1] Department of Orthopedic Surgery, Faculty of Medicine and Graduate of Medicine, Hokkaido University, Sapporo, Hokkaido, Japan

[2] Department of Advanced Medicine for Spine and Spinal Cord Disorders, Faculty of Medicine and Graduate of Medicine, Hokkaido University, Sapporo, Hokkaido, Japan

***Corresponding Author:**

Katsuhisa Yamada, MD, PhD, Department of Orthopedic Surgery, Faculty of Medicine and Graduate of Medicine, Hokkaido University, N15W7, Sapporo, Hokkaido 060-8638, Japan; Tel: 81-11-706-5934, Fax: 81-11-706-6054, E-mail: yka2q@yahoo.co.jp

Hideki Sudo, MD, PhD, Department of Advanced Medicine for Spine and Spinal Cord Disorders, Faculty of Medicine and Graduate of Medicine, Hokkaido University, N15W7, Sapporo, Hokkaido 060-8638, Japan; Tel: 81-11-706-5934, Fax: 81-11-706-6054, E-mail: hidekisudo@yahoo.co.jp

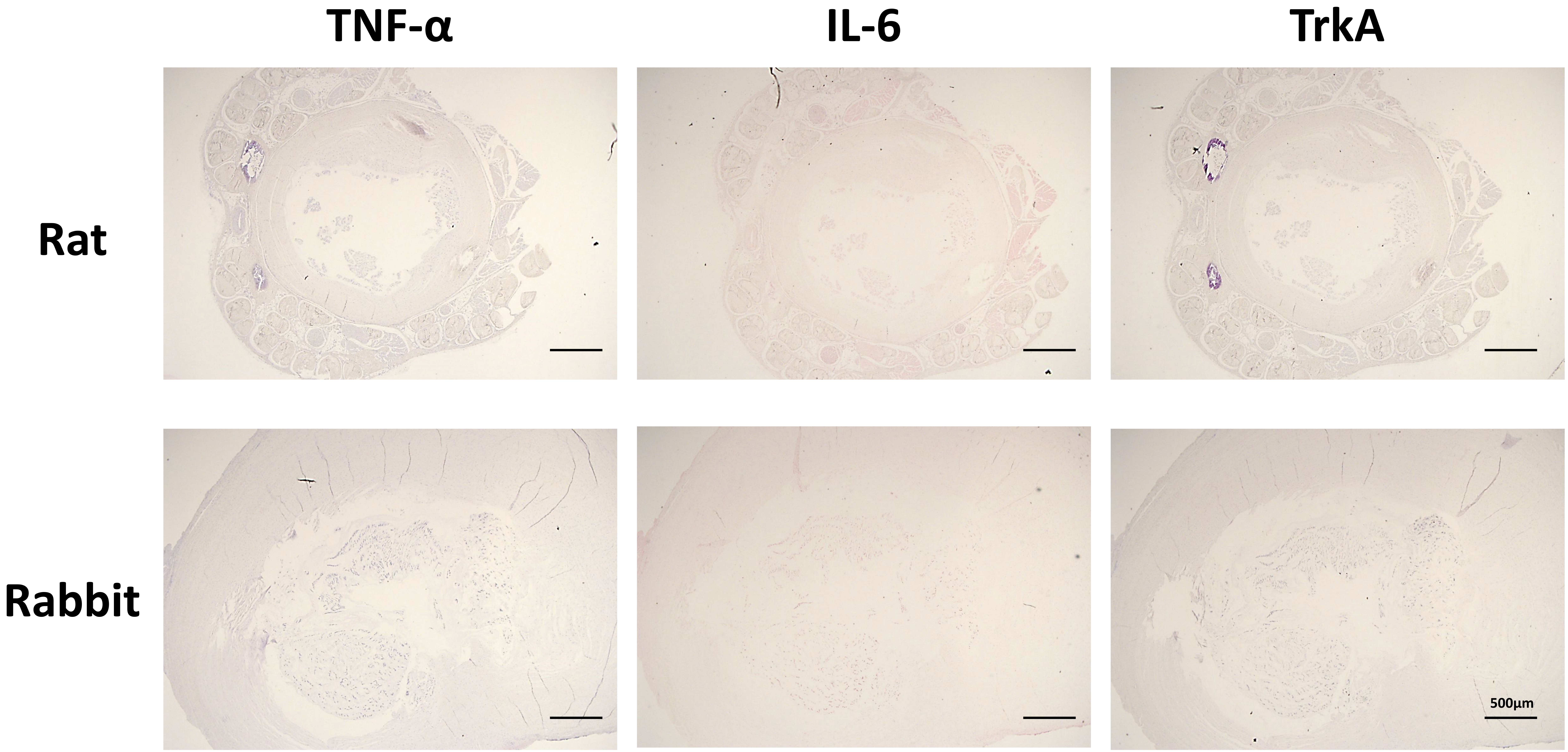

Supplementary fig. 1 Negative controls for TNF-a, IL-6 and TrkA in immunohistochemistry. TNF-α -positive cells are stained red and nuclei was stained purple using hematoxylin. IL-6 -positive cells are stained green and nuclei was stained red using nuclear fast red. TrkA -positive cells are stained brown and nuclei was stained purple using hematoxylin. Scale bar, 500 μm.

Supplementary Table 1. Histological grading

Category	Score		
	Sham	Punch	Gel
Endplate	0	0.67 ± 0.47	0.17 ± 0.37
AF	0	1	0.33 ± 0.47
Boundary between NP and AF	0	1.67 ± 0.47	0.17 ± 0.37
Cellularity of NP	0	2	0.5 ± 0.5
Matrix NP(H&E)	0	2	0.5 ± 0.5
Matrix NP(SO)	0	2	0
Total score	0	9.33 ± 0.94	1.67 ± 1.89

N = 6 discs in each group, mean ± standard deviation. NP; nucleus pulposus. AF; annuls fibrosus. H&E; hematoxylin and eosin. SO; safranin O
